# Exploring bioactive peptides from bacterial secretomes using PepSAVI‐MS: identification and characterization of Bac‐21 from *Enterococcus faecalis* pPD1

**DOI:** 10.1111/1751-7915.13299

**Published:** 2018-07-16

**Authors:** Christine L. Kirkpatrick, Nicole C. Parsley, Tessa E. Bartges, Casey E. Wing, Sushma Kommineni, Christopher J. Kristich, Nita H. Salzman, Steven M. Patrie, Leslie M. Hicks

**Affiliations:** ^1^ Department of Chemistry The University of North Carolina at Chapel Hill Chapel Hill NC USA; ^2^ Department of Pathology Southwestern Medical Center The University of Texas Austin TX USA; ^3^ Division of Gastroenterology Department of Pediatrics Medical College of Wisconsin Milwaukee WI USA; ^4^ Department of Microbiology and Immunology Medical College of Wisconsin Milwaukee WI USA; ^5^ Department of Chemistry Northwestern University Evanston IL USA

## Abstract

As current methods for antibiotic drug discovery are being outpaced by the rise of antimicrobial resistance, new methods and innovative technologies are necessary to replenish our dwindling arsenal of antimicrobial agents. To this end, we developed the PepSAVI‐MS pipeline to expedite the search for natural product bioactive peptides. Herein we demonstrate expansion of PepSAVI‐MS for the discovery of bacterial‐sourced bioactive peptides through identification of the bacteriocin Bac‐21 from *Enterococcus faecalis* pPD1. Minor pipeline modifications including implementation of bacteria‐infused agar diffusion assays and optional digestion of peptide libraries highlight the versatility and wide adaptability of the PepSAVI‐MS pipeline. Additionally, we have experimentally validated the primary protein sequence of the active, mature Bac‐21 peptide for the first time and have confirmed its identity with respect to primary sequence and post‐translational processing. Successful application of PepSAVI‐MS to bacterial secretomes as demonstrated herein establishes proof‐of‐principle for use in novel microbial bioactive peptide discovery.

## Introduction

Antibiotic‐resistant infections are widespread, constantly evolving, and one of the biggest threats to global health (World Health Organization, 2017). While multidrug resistance (MDR) is occurring across an alarming number of bacterial species, among the most threatening MDR gram‐positive organisms are enterococci. Although common commensal bacteria, enterococci are leading causes of hospital‐acquired infections and are becoming highly antibiotic resistant (Huycke *et al*., 1998; Kristich *et al*., 2014). Many species display intrinsic resistance to beta‐lactam antibiotics, and further acquired resistance to tetracyclines, rifampin and vancomycin, among others, severely limits therapeutic treatment options (Kristich *et al*., 2014; Miller *et al*., 2014). While the *Enterococcus* genus includes over 17 species, only a few have been shown to cause infections in humans. *Enterococcus faecalis* and *Enterococcus faecium* are the two most prevalent infectious species in humans and account for > 90% of human isolates (Olawale *et al*., 2011). It is difficult, if not impossible in some cases, to treat these emerging and highly resistant enterococcal strains. It is crucial to develop analytical tools and novel therapeutic strategies to both characterize and treat these deadly strains.

Many lactic acid bacteria, including enterococci, produce bacteriocins – small cyclic peptides capable of killing or inhibiting growth of related or similar species (Corr *et al*., 2007; Cruz *et al*., 2013; Grande Burgos *et al*., 2014; Perez *et al*., 2014). Bacteriocin production is one tool bacteria can use to gain a competitive advantage in an environment and establish a stable niche for the producing strain (Corr *et al*., 2007; Hibbing *et al*., 2010; Dobson *et al*., 2012). The enterococcal bacteriocin Bac‐21 is encoded for by the sex pheromone‐responsive conjugative plasmid pPD1 (Tomita *et al*., 1997). This class of plasmids constitutes a complex and highly efficient system used to both transfer genetic information and function as a regulator of bacterial virulence. To date, over 20 of these plasmids have been identified but only among enterococcal species (Wardal *et al*., 2010). The pPD1 plasmid is predicted to produce a mature, 70 amino acid bacteriocin, Bac‐21, identical in nucleotide sequence to another well‐characterized bacteriocin, AS‐48 (Tomita *et al*., 1997; Maqueda *et al*., 2004). However, the active Bac‐21 functional peptide has yet to be characterized. As demonstrated recently, the Bac‐21 producing enterococcal strain is able to specifically decolonize antibiotic‐resistant enterococci from the gastrointestinal tract (Kommineni *et al*., 2015). This has major implications as a novel therapeutic strategy to overcome difficult‐to‐treat enterococcal infections, and by extension a similar approach could be used to combat other problematic gut organisms. While this highlights potential therapeutic implementation of bacteriocins, we posit that these peptide toxins represent a large and relatively untapped source of bioactive peptides that can be harnessed for drug discovery applications.

PepSAVI‐MS (*S*tatistically guided bio*a*ctive *pep*tides prioritized *vi*a *m*ass *s*pectrometry) was developed to expedite the search for botanical bioactive peptides while addressing limitations of bioassay‐guided fractionation and genome mining approaches (Kirkpatrick *et al*., 2017). To enhance the search for potent and effective antimicrobial peptides (AMPs), we now extend this pipeline to bacterial secretomes, a rich source of AMPs with potentially novel mechanisms of action (MOAs) created and refined by extreme inter‐ and intra‐species competition (Riley and Wertz, 2002; Golneshin *et al*., 2015; Kommineni *et al*., 2015). Herein, we demonstrate expansion of PepSAVI‐MS to bacterial‐sourced AMPs using the bacteriocin Bac‐21 from *Enterococcus faecalis* harbouring the pPD1 plasmid. A robust agar diffusion assay approach was implemented to screen bioactive peptides against infectious enterococcal strains. Additionally, we experimentally validate the Bac‐21 peptide sequence and post‐translational processing for the first time. Successful application of PepSAVI‐MS to bacterial secretomes is demonstrated with this representative species and establishes proof‐of‐principle for use in novel microbial bioactive peptide discovery.

## Results and discussion

### Overview

**Figure 1:**
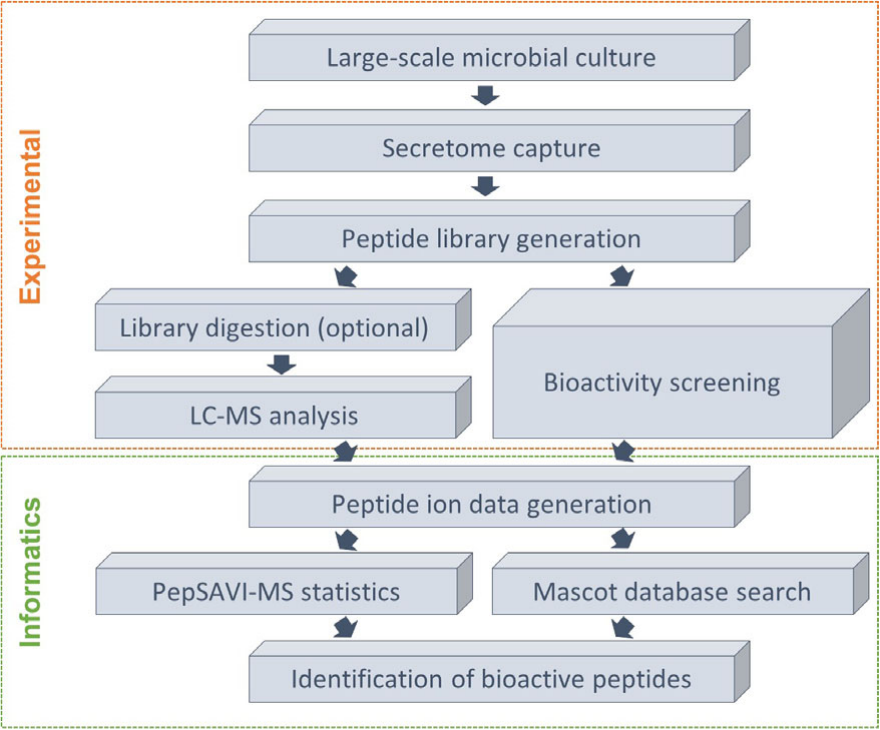
PepSAVI‐MS workflow for bacterial secretome analysis. Overall workflow for PepSAVI‐MS application to bacterial secretomes using digested peptide libraries. The developed workflow is amenable to large‐scale microbial culture growth to produce secreted peptides in sufficient quantities for downstream analysis. Secreted peptides are captured with cation exchange resin and crudely fractionated for creation of each peptide library. Peptide libraries are subject to LC‐MS analysis and bioactivity screening, and generated data sets are combined and informatically processed to identify bioactive peptides.

PepSAVI‐MS implements a multipronged approach for bioactive peptide discovery that utilizes selective extraction and fractionation of peptides from source material, bioactivity screening and mass spectrometry‐based peptidomics for the identification of putative bioactive peptide targets. PepSAVI‐MS was originally established for constitutively expressed peptides from botanical‐sourced species and was recently validated for fungal secretomes (Kirkpatrick *et al*., 2018). Now, we extend this pipeline to capture secreted peptides from bacterial sources (Fig. 1). Additionally, we demonstrate that digestion of source libraries post‐bioassay is an optional step for more facile detection of very large bioactive species (peptides/proteins) that may be difficult to detect intact depending on available LC‐MS/MS hardware. Expansion of PepSAVI‐MS to bacterial secretomes using digested peptide libraries highlights the versatility and wide applicability of this pipeline. Furthermore, using the secretome capture methods presented herein in conjunction with high‐resolution, high mass accuracy mass spectrometry, we are able to detect and characterize the active, mature Bac‐21 peptide for the first time.

### Secretome capture and peptide library generation

Dynamic microbe–microbe interactions and environmental competitive advantage are mediated by an array of secreted host‐defense peptides. Although created for survival, the innate bioactivity of these peptides poses a promising source of molecules to exploit for drug discovery. As such, we have expanded PepSAVI‐MS to capture potential bioactive components from the microbial secretome. Secreted peptides are collected from the cell‐free supernatant using weak cation exchange (WCX) resin added directly to the media and eluted in bulk using high salt. As the majority of bioactive peptides have been found to be highly positively charged, these peptides will be retained using WCX resin. However, other resin chemistries can easily be substituted at this stage for capture of a specific class of bioactive peptides with unique chemical properties. Concentrated elutions are then combined and crudely fractionated using SCX for peptide library creation.

### Bioactivity screening

**Figure 2:**
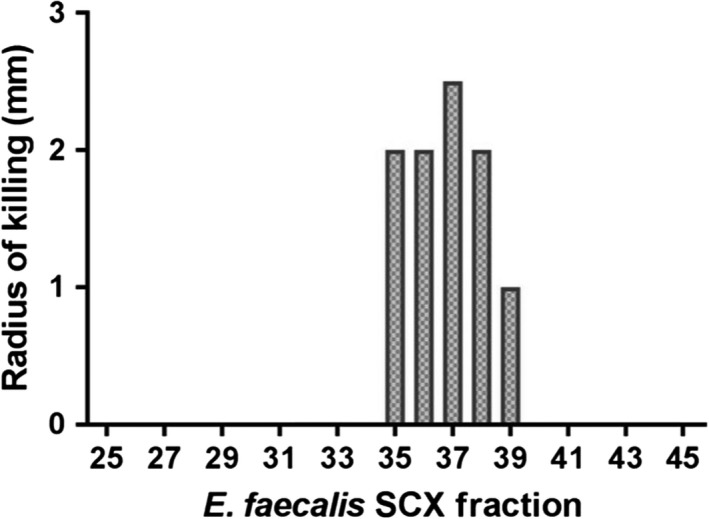
*Enterococcus faecalis* SCX bioactivity. *E. faecalis* pPD1 SCX fraction library bioactivity against *Enterococcus faecium* JL282. Assays were performed in duplicate, and the average radius of inhibition is plotted for each fraction.

PepSAVI‐MS is amenable to a variety of bioassay formats and can be modified to accommodate any target pathogen. High‐throughput microtitre‐based assays and agar diffusion‐based assays have been previously demonstrated with PepSAVI‐MS (Kirkpatrick *et al*., 2017, 2018). While many bacterial species bode well in high throughput 96‐well assays (as presented in the original implementation of the PepSAVI‐MS pipeline), this format is not amenable to some bacterial species that fail to grow to high density (e.g. < 0.5 OD) in low volume formats. This was the case with enterococcal cultures in Mueller Hinton Broth (MHB) and as such, agar‐based diffusion assays (originally developed for fungal cultures) were used to examine the activity of the *E. faecalis* peptide library against a susceptible *E. faecium* culture. The *E. faecalis* pPD1 peptide library shows strong activity against *E. faecium,* with measurable zones of inhibition present in fractions 35–39 (Fig. 2). The bioactivity region is a discrete and well‐defined profile spanning five sequential fractions with varying zones of inhibition. This representative profile is ideal for PepSAVI‐MS as the bioactive peptide responsible for the activity will have five paired bioactivity and LC‐MS abundance data points to facilitate statistics and modelling.

### MS profiling of E. faecalis pPD1 peptide library

The peptide library is subjected to LC‐MS/MS analysis to obtain accurate mass and peptide abundance data for all constituents. PepSAVI‐MS was originally demonstrated with top‐down, intact peptidome libraries. However, an optional digestion prior to LC‐MS/MS analysis may facilitate detection of target peptides in a manner similar to the bottom‐up Activity‐Correlated Quantitative Proteomics Platform (ACPP) used to identify enzymes with targeted reaction specificity (Ma *et al*., 2017). This approach is particularly useful for larger peptides with low charge density or poor ionization efficiency, or for investigations implementing resolving power‐limited mass spectrometry hardware. In such cases, database searching against the same or highly related microbial species can be used to determine the peptide constituents within each fraction and the proteolytic peptide(s) can be used for quantification and modelling via PepSAVI‐MS. Proteolytic peptides highly ranked as putatively bioactive can be used as a proxy for the intact peptide present in each fraction. This digestion step is demonstrated herein for the *E. faecalis* peptide library and is validated through comparison to the original top‐down intact analysis approach. Positively charged residues (i.e. Arg, Lys) frequently occur in bioactive peptides and would be cleaved by trypsin into many short peptides that elude LC‐MS detection. Thus, endoproteinase Glu‐C, which cleaves c‐terminal of glutamic acid residues, was used for proteolytic processing in order to obtain longer peptides with a higher probability to achieve increased protein coverage.

**Figure 3:**
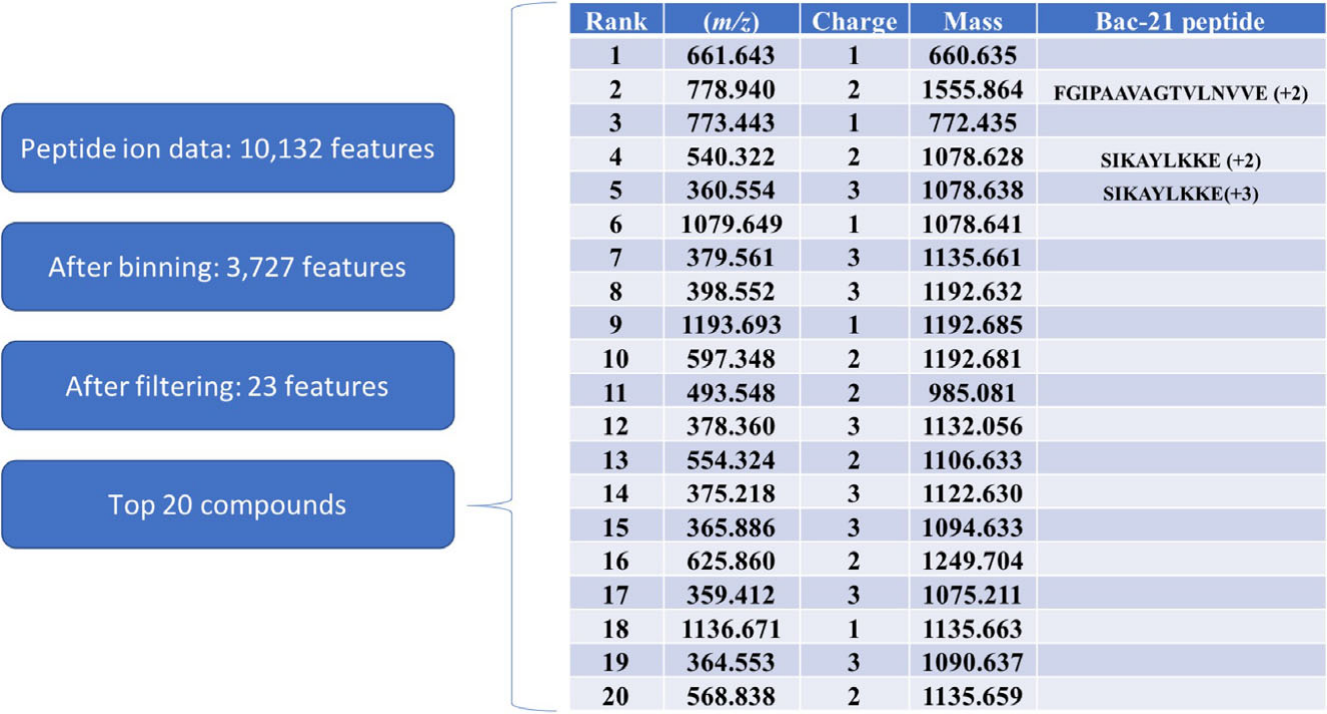
PepSAVI‐MS digested peptide library statistical modelling. PepSAVI‐MS statistical modelling summary, including the list of the top 20 compounds, denoted by rank, *m/z* ratio and charge state, to exit the PepSAVI‐MS statistical model for the digested peptide library. The first compound can be considered the most likely compound contributing to the observed bioactivity, with the likelihood decreasing down the rank list. Ranked peptides identified as Bac‐21 are noted.

**Figure 4:**
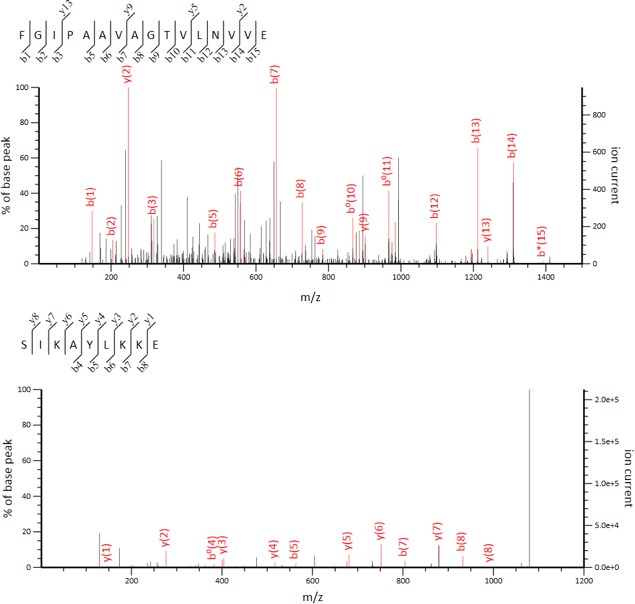
Mascot identification of Bac‐21 Glu‐C peptides. Representative MS/MS spectra generated from CID fragmentation of Bac‐21 Glu‐C peptides on a TripleTOF 5600 Q‐TOF platform. Peptide fragment maps used for Mascot identification of each statistically ranked Bac‐21 peptide are shown.

Peptide ion data reveals 10132 features detected across Glu‐C digested fractions 30–45 for the *E. faecalis* pPD1 library. This peptide ion data is used for both unbiased data reduction and statistical modelling with unassigned *m/z* ratios and database searching using Mascot for identification. For unbiased data reduction and statistical modelling, peptide ion data is first reduced to features most likely contributing to the bioactivity in each region through mass, retention time and elution profile mapping. When applied to the *E. faecalis* pPD1 data set, this reduction identified 3727 unique *m/z* ratios that were further filtered to 23 compounds with abundance profiles spanning the bioactivity region. Statistical modelling using elastic net penalized linear regression of this filtered data set reveals a candidate list of compounds likely responsible for the bioactivity region. After modelling, a list of the top 20 *m*/*z*'s likely contributing to the observed bioactivity is generated (Fig. 3). Then, comparison of the top 20 contributing *m/z* ratios with the Mascot output for the entire data set facilitates the assignment of top contributors. In this case, the 2nd, 4th and 5th top contributors (*m/z* ratios 778.94, 540.32, and 360.55) correspond to the +2 and +3 charge states of two Bac‐21 Glu‐C peptides: FGIPAAVAGTVLNVVE (+2) and SIKAYLKKE (+2, +3) (Figs 3 and 4). Glu‐C digestion of the *E. faecalis* pPD1 library yielded three of the four possible Bac‐21 peptides, providing 58% sequence coverage of the mature peptide. While three peptides were detected using Mascot, only two (FGIPAAVAGTVLNVVE and SIKAYLKKE) ranked in the top 20 compound list after statistical modelling. It is likely that low abundance and incomplete digestion were contributing factors to this result. Enzymatic digestion using proteases with higher digestion efficiency may increase the number of identified and ranked proteolytic peptides, increasing confidence in bioactive peptide identification in future experiments.

Analysis of intact *E. faecalis* pPD1 peptide library reveals 240 unique features across fractions 32–42. Binning and filtering parameters were adjusted for the increased size of intact peptides, but was modeled against the same bioactivity data as the Glu‐C digested peptide library. The resulting top 20 list after binning, filtering and modelling featured all three of the detected Bac‐21 charge states, ranking 2nd (+6), 14th (+4) and 17th (+5).

### Characterization of intact Bac‐21

**Figure 5:**
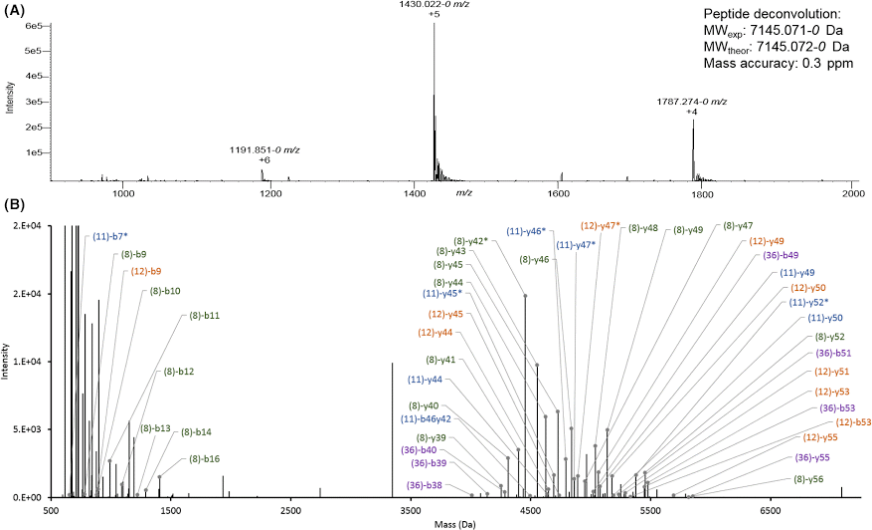
Characterization of intact Bac‐21.A. Mass spectrum of intact Bac‐21 (LTQ‐Orbitrap XL).B. Deconvoluted and annotated CID spectrum of 1192 *m/z* (Bac‐21 + 6 charge state). Fragmentation coverage was determined using the four most prevalent linear sequences, denoted by the number of the starting amino acid from the initial cyclic sequence. B‐ and y‐ions are labelled using traditional nomenclature with the number of the starting amino acid residue in parenthesis. Full linear sequences and ion coverage are provided below for clarity. Ions denoted with * indicate that another fragment identity is possible for that detected mass.

While evidence of *E. faecalis* Bac‐21 has been demonstrated through bioactivity (Kommineni *et al*., 2015), Bac‐21 has never been molecularly characterized. Hence, the most abundant Bac‐21 containing library fraction was characterized pre‐digestion (i.e. intact) using direct infusion, high‐resolution mass spectrometry in combination with collision induced dissociation for increased sensitivity and accurate mass detection. As intact Bac‐21 is a cyclic peptide, two fragmentation events must occur to produce fragment ions via MS/MS. The initial fragmentation is a ring opening event that can occur at any amino acid residue but will produce the same precursor mass. The second fragmentation event produces the classical b‐ and y‐ ion series typically associated with CID. While the initial fragmentation event produces identical linear masses independent of the initial cleavage point, the b‐ and y‐ions generated from each of these linear sequences will be unique to the initial cleavage point. As such, fragmentation of cyclic peptides exponentially increases the number of possible fragment ions produced from a single peptide. While providing a wealth of information to use for peptide sequencing, interpretation of these MS/MS spectra is often complex and confident fragment assignment is often ambiguous in many cases. Automatic software programs are crucial to facilitate this process, and an in‐house custom informatics search engine (Plymire *et al*., 2017) was used in this case. As such, the accurate intact mass of Bac‐21 was determined to be 7145.071*‐0* Da (7145.072*‐0* Da theoretical; 0.3 ppm mass accuracy) with 70% sequence coverage obtained via CID fragmentation of the +6 charge state (Fig. 5). Although less abundant than the +5 charge state, more fragment ions were generated from isolation and fragmentation of the higher charge state and thus the results from the +6 charge state are presented herein. These results indicate that Bac‐21 is identical in both primary protein sequence and post‐translational processing (head‐to‐tail cyclization) to the known bacteriocin AS‐48 (Tomita *et al*., 1997; Maqueda *et al*., 2004). Unsurprisingly, the most frequent initial fragmentation event occurred at the only proline residue (denoted as the 8th amino acid) in Bac‐21. The next, most frequent initial fragmentation events occurred at 11th, 12th and 36th amino acid residues of V, A and G, respectively, and likely indicate structural weak points in the folded peptide. As shown in Fig. 5, assignment of low mass fragment ions proved especially challenging. This is likely due to the occurrence of internal fragmentation events, as well as the presence of additional linear sequences contributing to the observed MS/MS spectra.

## Experimental procedures

### Microbial strains and growth conditions

*Enterococcus faecalis* (CK135) harbouring pPD1 (Yagi *et al*., 1983; Kristich and Little, 2012; Kommineni *et al*., 2015) and *E. faecium* (JL282) (Kristich and Little, 2012) strains were described previously. All *Enterococcus* strains were grown in Mueller Hinton Broth (MHB, BD Difco) and four 5‐ml starter cultures of *E. faecalis* were added to 2 l of MHB and were grown to late‐log phase at 37°C. Cells were removed by centrifugation (500 × *g* for 5 min), and the supernatant was collected.

### Creation of peptide libraries: Secretome capture

The cell‐free supernatant was adjusted to a pH of 5.5 and stirred overnight with 140 ml of CM Sephadex C‐25 resin hydrated in 25 mM sodium acetate, pH 5.5. Slurry mixture was gravity packed into a column and washed with two column volumes of 25 mM sodium acetate, pH 5.5, to remove unbound components. Peptides were eluted with 25 mM sodium acetate, pH 5.5, with 1 M NaCl and five 50‐ml fractions were collected. Fractions were buffer exchanged into 5 mM ammonium formate, pH 2.7, using 3 kDa spin concentration filters (Millipore) and concentrated 10 × . *Strong cation exchange fractionation*. Enterococcus crude fractions 2 and 3 (250 μl/fraction) were combined for strong cation exchange (SCX) fractionation via HPLC. The sample was subject to a 55‐min SCX method using a PolySulfoethyl A column (100 mm × 4.6 mm, 3 μm particles, PolyLC). A salt gradient was employed using a linear ramp of 5 mM ammonium formate, 20% acetonitrile, pH 2.7 to 500 mM ammonium formate, 20% acetonitrile, pH 3.0. Fractions were collected in one‐minute increments and desalted with three washes of 1.3 ml deionized water using a vacuum concentrator.

### Bioactivity screening

The susceptible culture selected for *E. faecalis* pPD1 killing assays, *E. faecium* JL282*,* was grown overnight in MHB. Soft agar was prepared for the agar diffusion assay by the addition of 1.5% bacto agar (BD) to broth solutions. Microbes were added to warm (48°C), soft agar and poured into 100 × 15 mm culture dishes. Once solidified, wells were carved in the agar and 50 μl of each test fraction was added to a different well. Plates were incubated at 37°C until visible growth inhibition was present (~2 days). Radial zones of clearance were measured around the point of application. Bacterial assays were performed in duplicate.

### LC‐MS/MS analysis of digested peptide library

The *E. faecalis* peptide library was subject to reduction, alkylation and Glu‐C digestion (*Staphylococcus aureus* Protease V8) prior to LC‐MS/MS analysis. Fractions were reduced using 10 mM dithiothreitol (30 min, 45°C, 850 rpm) and subsequently alkylated with 100 mM iodoacetamide (15 min, 25°C, 850 rpm) prior to overnight digestion with Glu‐C (37°C, 850 rpm). *E. faecalis* digested fractions were cleaned up with Pierce C18 zip tips (Thermo Fisher Scientific, Waltham, MA, USA) before subsequent LC‐MS analysis. Peptide libraries were analysed via a nano‐LC‐ESI‐MS/MS platform: Waters nanoAcquity UPLC coupled to an AB Sciex TripleTOF 5600. Peptide fractions were diluted to ~0.2 μg/μl and acidified to 0.1% formic acid. Five microlitres of each sample were injected onto a trap column (NanoAcquity UPLC 2G‐W/M Trap 5 μm Symmetry C18, 180 μm × 20 mm: Waters) before transfer to the analytical C18 column (10k PSI, 100 Å, 1.8 μm, 75 μm × 250 mm: Waters). Peptide separation was carried out at a flow rate of 0.3 μl/min using a linear ramp of 5–50% B (mobile phase A, 0.1% formic acid; mobile phase B, 0.1% formic acid in acetonitrile) over 30 min. The MS was operated in positive ion, high sensitivity mode with the MS survey spectrum using a mass range of 350–1600 *m/z* in 250 ms and information dependent acquisition (IDA) of MS/MS data, 87 ms per scan. For IDA MS/MS experiments, the first 20 features above 150 counts threshold and having a charge state of +2 to +5 were fragmented using rolling collision energy ±5%. Each MS/MS experiment put the precursor *m/z* on an 8‐second dynamic exclusion list. Auto calibration was performed every eight samples (8 h) to assure high mass accuracy in both MS and MS/MS acquisition. The mass spectrometry data have been deposited to the ProteomeXchange Consortium via the PRIDE (Vizcaino *et al*., 2016) partner repository with the data set identifier PXD009003 and 10.6019/PDX009003. Deisotoped peak lists for each fraction were generated using Progenesis QI for Proteomics software (Nonlinear Dynamics, v.2.0). Automatic processing settings were used to align all runs using fraction 37 as the reference and peak picking ions across the entire digested library. Identified features were quantified using AUC integration of survey scan data based on the summed intensity of each deisotoped feature. Data were exported as “peptide ion data” with the default parameters from Progenesis at the “Identify Peptides” stage in the software.

### Database searching of digested peptide library

Identification and location of Bac‐21 Glu‐C digested peptides was determined using Mascot (v.2.5.0; Matrix Science, http://www.matrixscience.com/). While the mature Bac‐21 peptide has until now not been physically detected or molecularly characterized, its identity in nucleotide sequence to another well‐characterized bacteriocin, AS‐48 was used to predict its protein sequence. Database searching was performed using the *Firmicutes* taxonomy of the SwissProt database (68 530 entries; accessed February, 2017) appended with the predicted peptide sequence of Bac‐21. Searches of MS/MS data used a Glu‐C protease specificity allowing two missed cleavages, peptide/fragment mass tolerances of 10 ppm/0.08 Da, a fixed modification of carbamidomethylation of cysteine residues, and variable modifications of acetylation at the protein N‐terminus and oxidation at methionine.

### Statistical modelling of digested peptide library

Areas of interest in the bioactivity profile were selected for subsequent data reduction and modelling. The bioactivity region for this data set was defined as fractions 35–39. Using the PepSAVI‐MS software package (https://cran.r-project.org/package=PepSAVIms) (Kirkpatrick *et al*., 2017), background ions were eliminated through retention time (14–45 min), mass (200–1600 for *E. faecalis* digest) and charge‐state (1–10, inclusive) filters to reduce the data to potential compounds of interest. For intact peptide libraries, singly charged species excluded to further select for highly positively charged bioactive peptides; however, these compounds are allowed to remain in the model for library digests. Retention time filters were selected to eliminate background ions, mass filters to select for the common mass range of bioactive peptides and charge state filters to eliminate unwanted small molecules. Peak‐picked data were binned and filtered using the previously established workflow‐informed criteria (Kirkpatrick *et al*., 2017). Briefly, binning was performed with a 0.05 Da window of features with identical charge states and filtering required a maximum abundance inside the bioactivity area of interest (fractions 35–39) with < 1% of that abundance outside of the chosen window. All features required minimum abundance of 100. All *m/z* species meeting these filtering criteria were modelled using the elastic net estimator with a quadratic penalty parameter specification of 0.001 to determine each species’ contribution to the observed overall bioactivity profile. The resulting list contains candidate compounds ranked in order of when they entered the model, such that the highest ranked compounds have the greatest likelihood of contributing to the bioactivity.

### LC‐MS analysis of intact peptide library

A select subset of fractions (32–42) including and surrounding the observed bioactivity region were subject to direct infusion on a Thermo Orbitrap Q Exactive HF‐X for intact mass analysis. Fractions were prepared in 50% water, 50% methanol and 0.1% formic acid with no dilution from the original library concentration and were injected at a flow rate of 5 μl/min. The mass spectrometer was operated at a resolving power of 120 000, positive ion mode, with 1000–2000 *m/z* range, and collecting 100 scans/sample. Progenesis QI for proteomics was used to generate a deisotoped peak list for intact samples, as described above.

### Statistical modelling of intact peptide library

Exported peptide ion data for the intact library were processed as described for the digested peptide library with the following adjustments: (i) binning was performed using a mass range of 2000–10 000 Da to account for intact peptides and (ii) a minimum intensity of 10 000 000 was required in the filtering stage to account for microscale direct infusion intensities. Exported peptide ion data contained 240 unique features, which were reduced to 177 after binning and 24 after filtering. The remaining 24 compounds entered to penalized linear regression model to determine the top 20 compounds most likely contributing to the observed bioactivity profile.

### Bac‐21 top‐down characterization

For top‐down analysis, the most abundant Bac‐21 containing *E. faecalis* pPD1 fraction was analysed on an LTQ‐Orbitrap XL platform (Thermo Fisher Scientific, Waltham, MA, USA). The sample was subject to direct infusion utilizing a 35 micron ESI emitter (New Objective Inc., Woburn, MA, USA). Samples were diluted to total peptide concentration of ~4 μM in 80% acetonitrile, 19% water, 1% acetic acid and injected at a flow rate of 0.5 μl/min. The mass spectrometer was operated at a resolving power of 30 000 at 400 *m/z*, positive ion mode, with 900–2000 *m/z* range. The spectra were deconvoluted using the AutoXtract algorithm in Protein Deconvolution 4.0 (Thermo Fisher Scientific, Waltham, MA, USA). CID fragmentation was performed on the +6 Bac‐21 charge state (1192 *m/z*) with a collisional energy of 35 V and 600–2000 *m/z* range. Data analysis was accomplished using a custom informatics search engine adapted from Plymire *et al*. (2017). The candidate sequences were tested against the fragmentation data at 15 ppm mass tolerance. The searches tested fragments against every possible initial cleavage event position for the cyclic peptide tested. Outputs report the number of fragment hits, rank initial cleavage sites by a Poisson‐based p‐score (Meng *et al*., 2001), and output maps associated with each position.

## Conclusion

PepSAVI‐MS is a highly versatile and easily adaptable pipeline for natural product bioactive peptide discovery. Successful application of PepSAVI‐MS for bacterial secretome capture demonstrates rapid and accurate identification of bioactive components from complex natural product secretomes and allows for focused downstream characterization and validation experiments on the most promising species. Expansion of PepSAVI‐MS to this historically rich source of bioactive molecules greatly enhances its capabilities and potential for novel bioactive peptide discovery. Furthermore, minor modifications to the original pipeline, including the use of agar diffusion assays for recalcitrant bacterial species and digestion of peptide libraries for sequenced organisms, facilitates exploration of bacterial secretomes and expands the applicability of PepSAVI‐MS to data acquired with lower resolution mass spectrometry hardware. Successful application of PepSAVI‐MS to microbial secretomes, as demonstrated with *E. faecalis*, opens the door to investigating microbial species with a new lens and stands to have positive implications in both human health and agriculture.

## Conflict of interest

None declared.
